# Neurophysiology of *Drosophila* Models of Parkinson's Disease

**DOI:** 10.1155/2015/381281

**Published:** 2015-04-20

**Authors:** Ryan J. H. West, Rebecca Furmston, Charles A. C. Williams, Christopher J. H. Elliott

**Affiliations:** ^1^Department of Biology, University of York, York YO1 5DD, UK; ^2^The MRC Protein Phosphorylation and Ubiquitylation Unit, The Sir James Black Centre, College of Life Sciences, University of Dundee, Dow Street, Dundee DD1 5EH, UK

## Abstract

We provide an insight into the role *Drosophila* has played in elucidating neurophysiological perturbations associated with Parkinson's disease- (PD-) related genes. Synaptic signalling deficits are observed in motor, central, and sensory systems. Given the neurological impact of disease causing mutations within these same genes in humans the phenotypes observed in fly are of significant interest. As such we observe four unique opportunities provided by fly nervous system models of Parkinson's disease. Firstly, *Drosophila* models are instrumental in exploring the mechanisms of neurodegeneration, with several PD-related mutations eliciting related phenotypes including sensitivity to energy supply and vesicular deformities. These are leading to the identification of plausible cellular mechanisms, which may be specific to (dopaminergic) neurons and synapses rather than general cellular phenotypes. Secondly, models show noncell autonomous signalling within the nervous system, offering the opportunity to develop our understanding of the way pathogenic signalling propagates, resembling Braak's scheme of spreading pathology in PD. Thirdly, the models link physiological deficits to changes in synaptic structure. While the structure-function relationship is complex, the genetic tractability of *Drosophila* offers the chance to separate fundamental changes from downstream consequences. Finally, the strong neuronal phenotypes permit relevant first *in vivo* drug testing.

## 1. Introduction

The discovery of inherited forms of Parkinson's disease (PD) provided a sea-change in our understanding of the disease. Importantly, new genetic animal models were created, based on the ever-expanding pool of PD-related pathogenic mutations. Commentators have noted that many mouse models have been disappointing, showing weak phenotypes [1, 2]. On the other hand, fly models have shown strong PD-related phenotypes, including reduced locomotion, loss of dopaminergic (DA) neurons, problems with reactive oxygen species, mitochondrial dysfunction, and protein aggregation [3]; for review see [4]. Fly models have been successful because the uniquely powerful genetic toolbox [5, 6], notably the GAL4-UAS system [7], has allowed tissue or neuron specific expression of dominant mutations (e.g., *α-synuclein*-*A30P* or* LRRK2-G2019S*) or RNA interference constructs (RNAi, e.g., against* parkin*). For recessive mutations, the toolbox facilitates generation of targeted point mutations or deletions. Additionally, the sharing of fly stocks and related reagents is common practice. A key outcome of this toolbox was the identification of common cellular effects across several PD-related mutations. For example, the “droopy wing” phenotype was instrumental in the discovery that* parkin* and* Pink1* interacted at the mitochondria [8, 9]. A number of other PD-related genes (including* Fbxo7* [10],* TRAP1* [11],* LRRK2* [12], and *α-synuclein* [13]) have since been implicated in this pathway suggesting a high degree of homology in the disease and fly model. Fly models have also linked  *α-synuclein* with Tau [14], extending the usefulness of* Drosophila* as a model. Furthermore, the fly models have begun to provide an* in vivo* testbed for drugs developed in biochemical or cell culture assays [15, 16].

Flies, like vertebrates, have DA neurons in the CNS [17]. The similarity between flies and vertebrates is also evident in the roles that dopamine plays in the fly CNS: it modulates locomotion, feeding, sleep/circadian rhythms, and learning [18]. However, relatively little is known about the physiological changes which occur in the nervous system when PD-related genes are manipulated. Almost all the neurophysiological evidence comes from analysis of recessive mutations in* Pink1* and* parkin*, and of the dominant gain of kinase function,* LRRK2-G2019S*. Our aim here is to review the evidence of nervous system dysfunction in these models, examining the changes in resting and synaptic potentials and linking these changes to behavioural deficits and loss of DA neurons. We also suggest how the combination of genetics and physiology in flies may provide novel insights into the progression of the excitotoxic neurodegenerative cascade.

Despite the small size of* Drosophila*, three physiologically tractable preparations are commonly used: the larval neuromuscular junction (NMJ), locomotory central pattern generator (CPG), and visual electroretinogram (ERG). All three preparations have been widely explored in wild-type* Drosophila* and in a range of non-PD settings. This provides a wealth of background information, which permits us to evaluate the impact of PD-mutations on the physiology of the motor, central, and sensory synapses.

## 2. The* Drosophila* Larval Neuromuscular Junction, a Model Synapse

**Figure 1:**
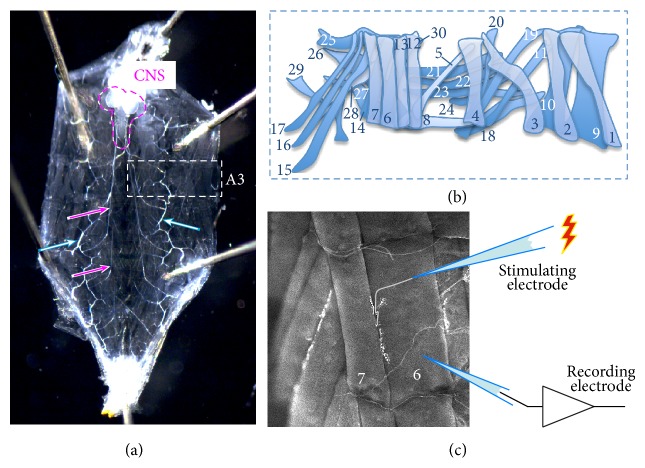
The consistent* Drosophila* larval NMJ. (a) Dissection of third instar* Drosophila* larvae reveals a distinct body plan, which is consistent between all larvae, comprised of 14 segments, 3 of which define the head and mouth region, 3 thoracic segments (T1–T3), and 8 abdominal segments (A1–A8; segment A3 indicated by the white dotted line). The CNS is outlined in magenta, the main nerve trunks indicated by magenta arrows. The trachea, which supply oxygen to the nerves and muscles remain intact, and two are indicated by the light cyan arrow. (b) Abdominal segments A2–A7 are composed of 2 bilaterally symmetrical hemisegments, each of which displays an archetypal structure comprising 30 distinct muscles. (c) These hemisegments have been extensively studied with a number of specific NMJs utilised as model synapses for a range of experimental purposes. Here we show muscle 6/7 (hemisegment A3). This muscle pairing has been extensively used for electrophysiological experiments due to the large size and accessibility of the NMJ, with the presynaptic motor neuron clearly visible innervating muscles 6 and 7. In this diagram we demonstrate how a suction electrode can be used to stimulate a single segmental nerve whilst recordings are made from muscle 6 (the larger of the pair).

The* Drosophila* larval NMJ is a well-characterised model synapse that has proved a highly amenable and successful tool to study synaptic development and neurotransmission [19]. In addition, the larval NMJ shows a significant degree of structural and functional similarity to vertebrate central synapses. For example, both vertebrate excitatory central synapses and* Drosophila* larval NMJs are glutamatergic and many of the molecules used in synaptic transmission are the same (see, e.g., [20] for details). However, in contrast to vertebrate central synapses, both the pre- and postsynaptic components of the larval NMJ are distinctly identifiable, accessible, and invariable from larva to larva, displaying archetypal structure and consistent neurophysiological responses (Figure 1). This means that NMJs can be easily compared between genotypes, providing a reliable model to investigate neurophysiological defects associated with disease causing mutations. This level of consistency would not be possible in vertebrate CNS synapses. Here we look to collate neurophysiological data, obtained using the larval NMJ as a model synapse, in studies looking at* Drosophila* mutants associated with Parkinson's disease.

## 3. NMJ Analysis Reveals Synaptic Perturbations in Parkinson's Mutant Flies

**Figure 2:**
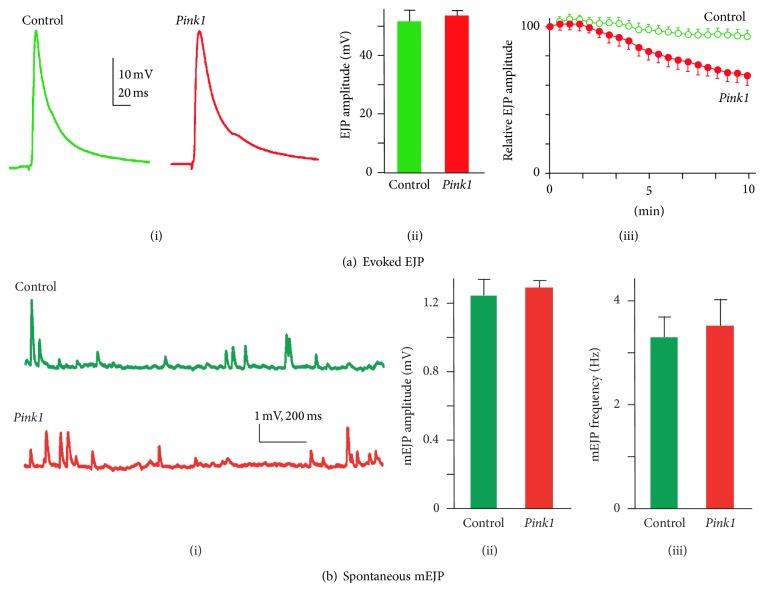
*Pink1* mutants display a progressive decline in synaptic transmission under high frequency stimulation. ((a)i-ii)* Pink1* (*Pink1* ^*B9*^) mutants showed no perturbation to basal neurotransmitter release properties, with no significant difference in the amplitude of evoked excitatory junction potentials (EJP) observed between* Pink1* mutants and controls. ((a)iii) Under high frequency stimulation (10 min, 10 Hz)* Pink1* mutants showed a substantial, progressive decline in synaptic transmission when compared to controls. ((b)i–iii)* Pink1* mutants showed no significant difference in spontaneous miniature EJP (mEJP) amplitude (i-ii) or frequency (i and iii) when compared to controls. Adapted from [21].

The most detailed analysis comes from the recessive, loss of function, mutation* Pink1* ^*B9*^ (Figure 2). These have a substantial progressive decline in synaptic transmission in response to high frequency stimulation at the larval NMJ [21]. However, there is no perturbation to basal release characteristics (i.e., no deficits in neurotransmitter release, spontaneous release frequency, or response amplitude) in this* Pink1* mutant. By using FM dyes to selectively label synaptic vesicle pools, it was demonstrated this decline resulted from a failure to mobilise the synaptic vesicle reserve pool. This phenotype could be, at least partially, rescued by administration of ATP to the synapse, supporting a role for* Pink1* in maintaining energy supply in periods of peak demand. A subsequent study supported this hypothesis, showing that* Pink1* had a role in the homeostatic regulation of mitochondria.* Pink1* modulated the activity of the electron transport chain, complex I [22]. In mouse, phosphoproteomic analysis in mouse revealed that* Pink1* nulls fail to phosphorylate mitochondrial complex I subunit NdufA10 (NADH dehydrogenase (ubiquinone) 1 alpha subcomplex 10) at Ser^250^. The consequences of this for synaptic transmission were explored in* Drosophila*, where the progressive decline in synaptic transmission seen in* Pink1* mutants was alleviated by expression of a phosphomimetic NdufA10. Additionally, phosphomimetic NdufA10 expression restored mitochondrial membrane potential and rescued both ATP synthesis and mobilisation of the synaptic vesicle reserve pool. The fly* Pink1* model has also proved useful in suggesting a novel therapeutic strategy, as the defects in synaptic mitochondrial membrane potential were rescued by feeding* Drosophila* on bacteria synthesising vitamin K_2_ [16].

**Figure 3:**
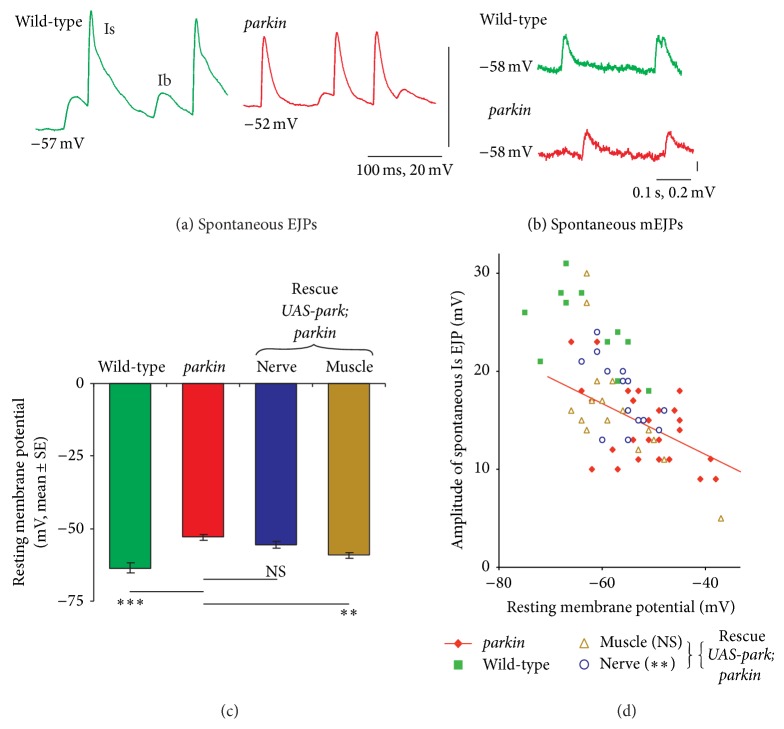
*Parkin* mutants display depolarisation of the muscle resting membrane potential coupled with reduced EJP amplitudes. (a) Spontaneous excitatory junction potentials (EJPs) were recorded from muscle 6 or 7. The mean resting potential and amplitude of the EJPs in the* parkin* mutant (*park* ^*Z3678*^/*parkin* ^*25*^) were ~12 mV more positive than in wild-type (*CS/w* ^*1118*^). In this recording both the Is and Ib EJPs are visible: both were reduced in the* parkin* mutant. (b) The spontaneous miniature EJPs (mEJPs) are smaller in the* parkin* mutant, as shown by these two recordings made at the same RMP. (c) Quantification of the change in resting membrane potential (RMP) in the* parkin* mutant, and the rescue by expression of* Drosophila* wild-type* parkin* in the muscle (*G14* GAL4), but not the nerve (*elav* ^*3e1*^ GAL4). (d) The plot of the size of Is EJP as a function of RMP shows that the more negative the RMP, the larger the EJP. However, at any chosen RMP, the wild-type EJP is bigger than those recorded from the* parkin* mutant. Note that all the green data points lie above the red regression line plotted through the* parkin* data. Expressing* parkin* in the nerves leads to data points that significantly lie above the red regression line, *χ*_1df_^2^ = 7.1, *P* < 0.01, showing effective rescue of the phenotype.* parkin* expression in the muscle leads to data distributed symmetrically about the regression line, no rescue. Data from [23] with (d) showing some data reanalyzed.

As with* Pink1*, mutations in* parkin* are associated with recessive, early-onset, and familial PD. In addition, genetic manipulations in the fly revealed interacting mitochondrial phenotypes in the muscle [8, 9]. As such it is not surprising that* parkin* mutant larvae, like* Pink1* larvae, also show perturbed synaptic transmission: a reduction of both evoked and spontaneous (mini) excitatory junction potential (EJP) amplitudes, along with depolarisation of the resting muscle membrane potential [23] (Figure 3). Reduced synaptic transmission is likely the result of impaired glutamate release, potentially due to the observed changes in synaptic morphology and/or ATP depletion. This in turn further alludes to an impairment of aerobic respiration in mutants implicated in Parkinson's disease.

Since the* G2019S* mutation in* LRRK2* is the most common cause of late-onset PD, two groups have examined the impact of overexpressing* LRRK2-G2019S* on the fly NMJ. Lee et al. [24] compared pre- and postsynaptic expression of the normal human form* hLRRK2* with the pathogenic human* LRRK2-G2019S* transgene at the larval NMJ. They demonstrated little effect postsynaptically with no significant alteration in any of the parameters measured (mEJP frequency, amplitude, quantal content, or EJP amplitude). In contrast, presynaptic expression elicited a significant increase in mEJP frequency, coupled with a reduced quantal content. Matta et al. [25] also examined the effect of* LRRK2-G2019S* overexpression at the NMJ, using FM dyes to show that ubiquitous expression impeded synaptic vesicle endocytosis.

The fly homolog to* LRRK2* is known as* Lrrk*. The homozygous loss-of-function* Lrrk* mutant also presents with deficits in synaptic transmission, characterised by a significant depletion in EJP amplitudes [24]. This phenotype was partially rescued by presynaptic, but not postsynaptic, expression of* Lrrk*. Nonetheless, mEJP amplitudes showed no difference to wild-type, implying a decreased quantal content.* Lrrk* mutants also displayed a significant increase in mEJP frequency. In a second study, using a different* Lrrk* loss-of-function mutant, no difference in EJP amplitude was found between* Lrrk* loss-of-function mutants and wild-types at baseline conditions, and mEJPs were larger [25]. This discrepancy in synaptic physiology may be due to the fact that different* Lrrk* mutant alleles were used by the two groups or may be due to differences in genetic background, as previous studies on* Lrrk* mutants have reported different lifespan phenotypes [26–28].

An important advance was derived from the observation that* Lrrk* mutants showed a progressive rundown with repetitive stimulation, indicating a link to defective synaptic vesicle cycling and enlarged vesicle structures within the synapse at an ultrastructural level [25]. This was due to LRRK phosphorylating endophilin-A (EndoA) at serine 75 in order to modulate EndoA dependent membrane deformation and endocytosis of the synaptic membrane. This was confirmed when heterozygous loss-of-function* EndoA* mutants rescued the progressive decline in synaptic transmission observed in* Lrrk* mutants during high frequency stimulation. Similarly presynaptic overexpression of EndoA could potentiate reduced synaptic membrane endocytosis observed using FM1-43.

A recent study of the dominant gene, *α-synuclein*, reported a similar phenotype as a result of presynaptic overexpression of wild-type human *α-synuclein*, eliciting enlarged synaptic vesicles and elevated mEJP amplitudes [29]. This was reverted by expression of the endosomal recycling factor Rab11. This data is of considerable interest, as flies do not have a close *α-synuclein* homolog but clearly respond to expression of human *α-synuclein*.

In addition to the study of synaptic transmission, the* Drosophila* larval NMJ has proven highly amenable and successful in the study of synaptic growth and development. For example, perturbed NMJ morphology and growth have been observed in numerous models of neurodegenerative diseases, including Alzheimer's and frontotemporal dementia as well as PD [24, 30, 31]. However, analysis of PD-related mutants reveals NMJ growth phenotypes do not consistently correlate with synaptic transmission phenotypes observed. For example, whilst* Lrrk* mutants and presynaptic expression of the pathogenic* LRRK2-G2019S* transgene both elicit a reduced quantal content and increased mEJP frequency,* Lrrk* mutants display a synaptic overgrowth phenotype and the* LRRK2-G2019S* NMJs are significantly undergrown [24]. Similarly, whilst both pre- and postsynaptic expression of* LRRK2-G2019S* elicited synaptic undergrowth, only presynaptic expression perturbed synaptic transmission. The observation of such differences between genotypes may allude to different molecular mechanisms implicated in pathology and, thus, may provide greater insight into the disease than by looking at synaptic transmission or NMJ morphology alone.

## 4. Examination of Rhythmic Patterns Reveals CNS Defects in Parkinson's Mutant Flies

Rhythmic movements depend on patterned output from the CNS to the motoneurons and thence to the muscles. A similar pattern usually persists when sensory input is reduced, though often at a lower frequency [32]. This led to the idea of a central pattern generator (CPG). These have been directly demonstrated by isolating the CNS in a dish of saline and recording the rhythmic pattern in a number of systems, both vertebrate and invertebrate [33]. The persistence of a “fictive crawling” pattern when sensory input was reduced in* Drosophila* larvae [34–36] has suggested that their locomotion depends on a CPG. The full details of the larval crawling CPG remain to be elucidated, but the motoneurons, glutamatergic, and cholinergic interneurons contribute [37] along with contributions from neurons expressing the clock gene* period* [38].

**Figure 4:**
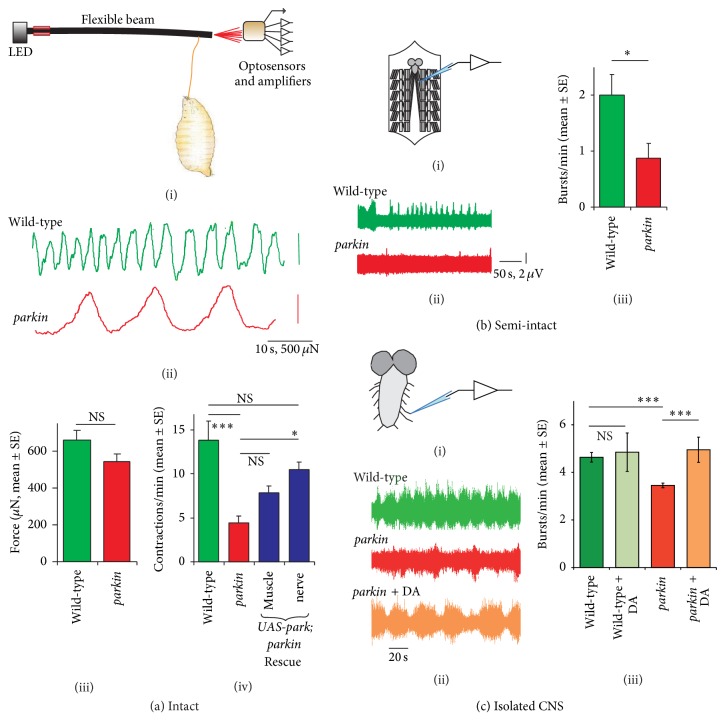
The* parkin* mutation slows the locomotor CPG, with rescue by neuronal expression of* parkin* or application of DA to the CNS. (a) Recordings with a larval extensometer (i) show that (ii–iv) the frequency (controlled by the CNS) but not the amplitude (controlled by the muscle performance) is affected by the* parkin* mutant (*park* ^*Z3678*^/*parkin* ^*25*^). The contraction frequency is rescued by expression of* Drosophila* wild-type* parkin* in the nerve (*elav* ^*3e1*^ GAL4), but not the muscle (*G14* GAL4). (b) In a semi-intact preparation, with en-passant nerve recordings, the frequency of bursting is reduced in the* parkin* mutant. (c) In the isolated CNS, the fictive locomotion rhythmic bursting pattern is also reduced in the* parkin* mutant and rescued by application of dopamine (DA, 1 mM in HL-3 saline supplemented with 3 mM ascorbic acid to reduce oxidation). Data in (a) and (b) from [23], with original recordings in (c).

In* parkin* mutants, the speed of locomotion was slowed by ~30% [23]. Using a larval extensometer, it was shown that this was due to a reduction in the frequency of peristalsis, while the strength of the contractions remained unchanged (Figure 4). Restoring* parkin* to the nervous system rescued the frequency of contractions. In a semi-intact preparation, the segmental nerves were severed, and the frequency of bursts of action potentials was recorded in the motoneurons' axons. The* parkin* mutants showed a 50% reduction in bursting rate. All this implies the* parkin* defect in these young* Drosophila* is principally in the CNS and that the muscles are not yet dysfunctional. In adult* parkin* flies, muscle degeneration gradually develops, and the flies become unable to maintain their normal wing posture [39].

In order to test the hypothesis that neural dysfunction precedes muscle degeneration, we have recorded from the motoneuronal axons after isolating the CNS. These recordings still show bursts of action potentials, though the frequency of these bursts is much lower than the frequency of peristaltic waves in the intact wild-type larva. Importantly,* parkin* mutants show a 30% reduction compared to wild-type controls (Figure 4). Application of 1 mM dopamine to the bath restored the bursting pattern of* parkin* nerves to wild-type levels but did not affect wild-type larvae. The frequency of the compound action potentials within the burst is not affected by the* parkin* mutations, suggesting the motoneurons are firing at a similar rate. We conclude that while* parkin* may reduce synaptic potentials generated from (and between) interneurons, it is possible that* parkin* depolarises the motoneurons (as it does the muscles), so compensating for changes in the interneuronal output.

## 5. Examination of Electroretinograms Reveals Synaptic Defects in Parkinson's Mutant Flies

Although still generally described as a movement disorder, PD is a complex multisystem disorder with patients experiencing a range of symptoms with both motor and nonmotor features. For example, a variety of visual associated defects ranging from dry eyes to abnormal light adaptation and complex hallucinations have all been reported in patients with PD [40]. Through the use of tyrosine-hydroxylase staining amacrine cells in the human retina have been identified as dopaminergic [41]. Retinal dopamine can be reduced in PD patients [42]. Therefore, some of the visual consequences of PD may originate in the retina, where dopamine is known to play a major role in signal regulation [43, 44].

At first sight, flies eyes seem quite unlike those of vertebrates. However, using silver staining, Cajal and Sanchez demonstrated that many of the neuronal circuits in the vertebrate and fly eye are fundamentally conserved [45]. This has since been corroborated using more advanced cytochemical approaches [46]. Of importance, like vertebrates, flies also have DA neurons in their visual system [47, 48] and DA circuits modulate fly vision [49–51].

Strong overexpression of some PD-related transgenes in the retina leads to developmental abnormalities, including *α-synuclein* [3, 13],* LRRK2* [52], or tau [14]. To test for abnormal physiology within the visual system, flash electroretinograms (fERGs) could be readily deployed. The anatomy of the fly eye makes it relatively easy to record fERGs; thus, the* Drosophila* ERG has been utilised for over 50 years, proving to be highly important in the characterisation of many of the key genes involved in phototransduction and the identification of over 200 ERG-defective mutants [53, 54]. However, we are not aware of physiological studies of these systems.

Hindle et al. [49] adopted an alternate approach: rather than using strong expression in the retina, they expressed the dominant* LRRK2*-*G2019S* mutation in just the DA neurons (*DA → G2019S*) and then used fERGs to analyse visual neurophysiology. The key result is that dopaminergic expression of* LRRK2-G2019S* leads to a reduction in all components of the fERG at 28 days old; no loss of response is seen with dopaminergic expression of the wild-type* hLRRK2* gene. The decline in visual function is sensitive to the* G2019S* mutation as dopaminergic expression of other mutations within the* LRRK2* gene, known to be pathogenic or to segregate with PD, shows no significant reduction in the fERG amplitude. Using other GAL4 drivers to express* LRRK2-G2019S* ubiquitously or in specific tissues of the eye, including the photoreceptors or lamina neurons, shows that the visual decline is specific for the expression of* LRRK2-G2019S* in DA neurons.

Whilst externally the eyes of* DA → G2019S* flies appear normal, the functional decline in vision of these flies is accompanied by anatomical neurodegeneration throughout the visual system. This includes disorganised retinas and frequent vacuoles appearing in the second- and third-order visual neuropils (lamina and medulla) [49]. Antibody staining reveals an increase in autophagy and apoptosis around the microvilli of the photoreceptors of old* DA → G2019S* flies. Electron micrographs show that the photoreceptor mitochondria of these flies become fragmented, swollen, and the cristae wider. DA neurons innervating the optic system were unperturbed in aged* DA → G2019S* flies suggesting that the loss of visual function and degeneration of the photoreceptors precede any loss of dopaminergic innervation of the visual lobes.

Increasing the demands on the visual system either through keeping flies in a pulsating light incubator or genetically through the introduction of the electrical-knock-in (EKI) transgene into the DA neurons to make them more active accelerates the decline in visual function due to* G2019S* expression [49].

The accelerated degeneration of the visual system through increased neuronal activity led to the hypothesis that young* DA → G2019S* flies could have amplified neuronal responses compared to wild-type flies. To test this hypothesis the steady-state visually evoked potential (SSVEP) method used in human visual electrophysiology was successfully translated to flies [15]. This technique is more sensitive than the widely used fERG because responses to many stimulus events are averaged together and out-of-band noise is eliminated from the analysis.

**Figure 5:**
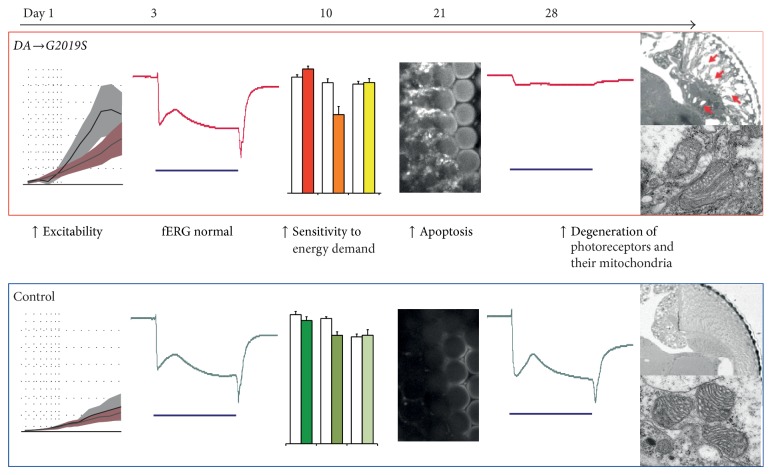
An excitotoxic cascade is initiated by the dopaminergic expression of the human* LRRK2-G2019S* transgene (*DA → G2019S*). In young, 1-day-old* DA → G2019S* flies, the eyes show much greater contrast sensitivity than the controls. At 3 days, the visual sensitivity measured by a flash electroretinogram (fERG) is indistinguishable from control flies. At 10 days, the* DA → G2019S* flies are more sensitive to energy demands than controls. Energy demand is increased by either treatment with flashing light or knockdown of potassium channels. By 21 days, the* DA → G2019S* eyes show evidence of apoptosis (anticleaved-caspase-3 staining). Old flies, 28 days, show little photoreceptor response, extensive vacuoles, and deformed mitochondria, showing loss of both function and structure. Data and exact genotypes in [15, 49].

One day after eclosion,* DA → G2019S* flies had a dramatically increased contrast sensitivity compared to controls expressing the wild-type* hLRRK2* or to those not expressing any transgene in their DA neurons [15]. The increased sensitivity of the* DA → G2019S* flies is thought to originate in the photoreceptors and is inherited by the second-order lamina neurons. These results, taken with Hindle's observations, suggest that a period of hyperactivity of the visual system occurs in young* DA → G2019S* flies. This starts an excitotoxic cascade (Figure 5), in which the flies soon start to suffer from an increased sensitivity to energy demand, followed by a cascade of degenerative events including apoptosis and autophagy. In old flies, the photoreceptors and their mitochondria have degenerated to such a degree that the vision of these flies is severely defective.

A key question is whether compounds that rescue* G2019S* phenotypes in cellular or* in vitro* assays also work* in vivo*. As this mutation occurs in the kinase domain of* hLRRK2*, flies were fed on inhibitors targeted at* LRRK2-G2019S*. One of these compounds, LRRK2-IN-1, has previously been identified as a* LRRK2* kinase inhibitor [55]. The second compound was a novel LRRK2 inhibitor, BMPPB-32 [15]. Both LRRK2-IN-1 and BMPPB-32 rescue the initial hyperactivity seen in young flies expressing* G2019S* in their DA neurons: the photoreceptor and neuronal responses are rescued to levels comparable to control flies. To test for off-target effects, the SSVEPs of flies with no* Lrrk* were measured. When LRRK2-IN-1 is given to these flies the SSVEP responses are significantly increased indicating that* in vivo* as* in vitro* LRRK2-IN-1 is binding to other kinases. When BMPPB-32 is applied there are no significant changes of the* Lrrk* mutant flies suggesting this compound does not have severe off-target effects and may be a promising candidate for future drug trials.

**Figure 6:**
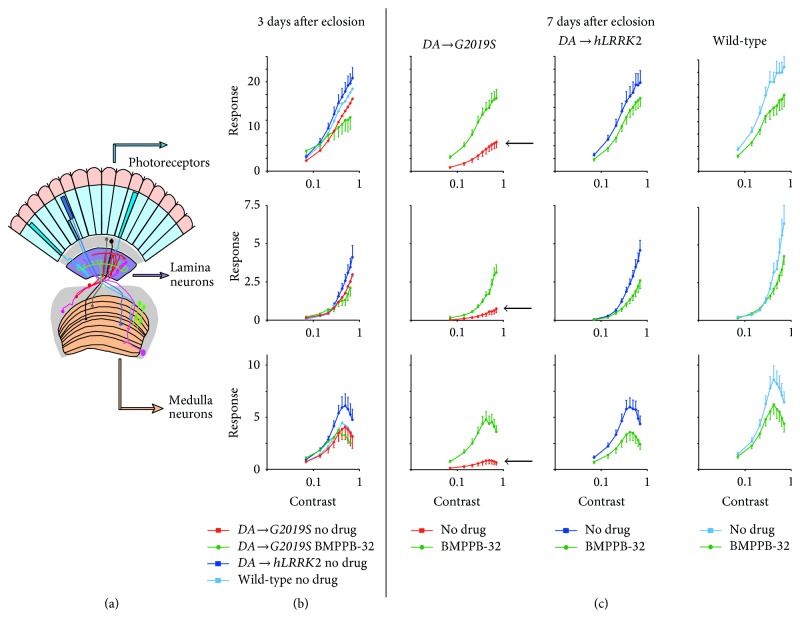
Rescue of dopaminergic* LRRK2-G2019S* visual neurophysiology by BMPPB-32 only applied during adult life. (a) Diagram of the fly eye, showing the lens (red), photoreceptors (blue), second-order lamina (purple), and third/fourth-order medulla (orange). Dopaminergic neurons are shown in green. When illuminated with flickering blue light, the overall (field) potential we record is separated by the fast Fourier transform (FFT) into components corresponding to the photoreceptor, lamina, and medulla neurons, respectively, details in [15]. (b) Larvae were raised on drug free food and some transferred to food containing 2.5 *μ*M of the specific* LRRK2* kinase inhibitor BMPPB-32 [15] on the first day of adult life. After 3 days, the contrast/response function (CRF) of flies expressing* LRRK2-G2019S* in the dopaminergic neurons (*DA → G2019S*) is indistinguishable from flies expressing* hLRRK2* or to those not expressing any transgene. BMPPB-32 application has no effect. (c) At 7 days, the* DA → G2019S* flies show very much reduced CRFs, well below the controls. BMPPB-32 restores the visual function to within 30% of the controls. There is no statistically significant effect of this drug on the control genotypes. Genotypes as in [15]; data not previously published.

Previously we tested these drugs by applying them throughout the entire lifespan (larva and fly). We have now extended this data by applying BMPPB-32 only after the time point where we see a neurophysiological phenotype, the start of adult life (Figure 5). This mimics the situation of a PD patient, who may only wish to start taking drugs once symptoms become apparent. In this experiment, larvae were raised on drug-free food, and the adult flies were given the drug on the day of eclosion. The adults were kept in a pulsating light incubator for 7 days. Already, the visual physiology of the* DA → G2019S* flies was severely reduced (Figure 6) with marked loss of the photoreceptor response (down by 70%). The reduction in signalling in the second- and third-order neurons was even more severe (lamina: 85%; medulla: 90%), suggesting a key synaptic phenotype. Control flies had visual responses indistinguishable from the younger, 3-day-old, flies. Feeding BMPPB-32 significantly improves the visual function of 7-day-old* DA → G2019S* flies, restoring the visual response to ~70% of control flies (Figure 6). Although the mean visual response in the control flies fed with BMPPB-32 appears slightly lower than the drug free controls, this difference is not statistically significant.

More recently, we have also shown that a second drug, UDCA (ursodeoxycholic acid), which rescues mitochondrial function in* LRRK2-G2019S* fibroblasts [56], also ameliorates the* DA → G2019S* visual neurophysiological deficit [57]. The impact of providing BMPPB-32 or UDCA to* DA → G2019S* flies after a phenotype has started to develop suggests that drugs like this may provide a disease modifying therapy as well as a preventative therapy. As UDCA is already licensed for liver disease, this is an exciting development.

## 6. Conclusion

Our analysis shows that the manipulation of PD-related genes in flies has revealed deficits in motor, CNS, and sensory synaptic signalling. Whilst relatively few groups are currently investigating these neurophysiological deficits, and thus some findings await independent confirmation, the observations remain striking. Furthermore, given the neurological impact of such mutations in the human population, we see four unique opportunities provided by the fly neurophysiology.

Firstly, these phenotypes are instrumental in exploring the links between common physiological problems (e.g., vesicular signalling, sensitivity to energy supply) and neurodegeneration. These are leading to the identification of plausible cellular pathways (and perhaps also possible partners, e.g., endophilin-A, NdufA10, Rab11). Flies provide a major opportunity here to separate the normal interactions in neuronal and synaptic function from generic cellular effects. The similarity in neuronal systems between fly and human, coupled with the observation of dopaminergic phenotypes in the fly, offers the potential to identify mechanisms that make DA neurons more sensitive to PD-related mutations.

Secondly, the models show a noncell autonomous signalling within the nervous system. This is most evident in the visual* LRRK2-G2019S* model, where expression of the transgene in the DA neurons affects the histaminergic photoreceptors, and the nondopaminergic second- and third-order (lamina and medulla) neurons. Other examples of nonautonomous signalling have been reported in the* Lrrk* and* parkin* mutants. These models therefore offer the opportunity to develop our understanding of the mechanisms by which pathogenic signalling expands, for example, by exosomes, and phenocopy Braak's view of the gradual spread of pathology in PD [58].

Thirdly, the physiological data we have reviewed are often linked to changes in synaptic structure. This is seen in the* parkin* and* Lrrk* mutants, and this may be related to oxidative stress. While the link between structure and function is complex, the genetic tractability of* Drosophila* offers the chance to use epistatic shielding to determine which changes (in anatomy/physiology) are fundamental and which are downstream consequences.

Finally, the strong neuronal phenotypes have also permitted the development of drug testing* in vivo*. This has been seen in both recessive and dominant genetic models, with tool compounds providing successful preventative and possibly disease modifying therapies.
